# Inflammatory and neutrophil extracellular trap markers to predict cardiac events after ST-segment elevation myocardial infarction

**DOI:** 10.1371/journal.pone.0319759

**Published:** 2025-04-01

**Authors:** Ana Blasco, Axel Rosell, Raquel Castejón, Ana Royuela, Charlotte Thålin, Elvira Ramil, Silvia Elorza, María-José Coronado, Paloma Martín, Javier Vázquez, Carolina González-Andrés, Juan M. Escudier, Javier Ortega, Carmen Bellas

**Affiliations:** 1 Cardiology Department, Hospital Universitario Puerta de Hierro-Majadahonda, Madrid, Spain; 2 Research Ethics Committee, Instituto de Investigación Puerta de Hierro-Segovia de Arana, Madrid, Spain; 3 Hematology and Regenerative Medicine, Department of Medicine Huddinge, Karolinska Institutet, Stockholm, Sweden; 4 Department of Clinical Sciences, Karolinska Institutet Danderyd Hospital, Stockholm, Sweden; 5 Internal Medicine Department, Hospital Universitario Puerta de Hierro-Majadahonda, Madrid, Spain; 6 Biostatistics Unit, Instituto de Investigación Puerta de Hierro-Segovia de Arana, Madrid, Spain; 7 Center for Biomedical Research in Epidemiology and Public Health Network (CIBERESP), Madrid, Spain; 8 Sequencing and Molecular Biology Unit, Instituto de Investigación Puerta de Hierro-Segovia de Arana, Madrid, Spain; 9 Clinical Biochemistry Department, Hospital Universitario Puerta de Hierro-Majadahonda, Madrid, Spain; 10 Confocal Microscopy Unit, Instituto de Investigación Puerta de Hierro-Segovia de Arana, Madrid, Spain; 11 Molecular Pathology Laboratory, Instituto de Investigación Puerta de Hierro-Segovia de Arana, Madrid, Spain; 12 Center for Biomedical Research Network (CIBERONC), Madrid, Spain; Massachusetts General Hospital - Harvard Medical School / Epidemiology Department - Harvard School of Public Health, UNITED STATES OF AMERICA

## Abstract

**Background and aims:**

Inflammation plays a pivotal role in the pathophysiology of ST-elevation myocardial infarction (STEMI). This involves neutrophil activation and the local release of pro-inflammatory mediators. The formation of neutrophil extracellular traps (NETs) in coronary thrombosis has been linked to poor short-term prognosis following STEMI, but the usefulness of specific circulating NET components as prognostic markers is unclear. We aimed to evaluate the NET-specific marker nucleosomal citrullinated histone H3 (H3Cit-DNA) and other classical inflammatory markers to predict adverse events after STEMI.

**Methods:**

This is a single-center retrospective cohort study of patients with STEMI undergoing primary percutaneous coronary intervention (PCI) from 2015 to 2019. We analyzed the association between serum H3Cit-DNA levels, double-stranded DNA, and classical inflammatory markers –such us interleukin (IL) 6 and 1β, TNF-α, and C-reactive protein (CRP)– on admission and the occurrence of major cardiovascular events (MACE), including death, reinfarction, urgent revascularization, or heart failure, after STEMI.

**Results:**

A total of 487 patients were studied, of which 380 were men [78%]; mean [SD] age of patients was 63 [13] years, and median [95%CI] follow-up was 5.4 [5.2-5.5] years. Median [IQR] H3Cit level was 179.30 [105.30-281.47] ng/ml. No relevant association was found between H3Cit-DNA levels and 30-day mortality (OR, 1.03 [95%CI, 0.71-1.50], p = 0.861) or MACE (0.98 [0.72-1.32], p = 0.879), Killip class (0.95 [0.74-1.21], p = 0.664), or left ventricular ejection fraction (ref.cat. > 50%; < 35%, RRR 1.01 [95%CI, 0.74-1.38], p = 0.952; 35-50%, 1.26 [1.07-1.48], p = 0.005]. Adding CRP and IL-6 levels as covariates to a model based on classical risk factors significantly improved the prediction of MACE at 30 days after STEMI (IDI 0.13; NRI 0.32, p < 0.05).

**Conclusions:**

Circulating levels of the NET marker H3Cit-DNA at the time of primary PCI were not predictive of cardiovascular events following STEMI. In contrast, the classical inflammatory markers CRP and interleukin-6 significantly enhanced the discriminative capacity of a clinical 30-day risk prediction model. These findings suggest that measuring circulating NET-specific markers may have limited utility in assessing the inflammatory state during the early stages of STEMI.

## Introduction

Inflammation plays a pivotal role in the pathophysiology of atherosclerosis. In ST-elevation myocardial infarction (STEMI), the initial inflammatory response is typically triggered by plaque rupture and coronary occlusion at the culprit lesion site. This process encompasses the activation of polymorphonuclear neutrophils (PMN), the recruitment of monocytes, and the local release of pro-inflammatory mediators, such as interleukins (IL) [1].

The acute cytokine surge enhances neutrophil activation, promotes the release of granule enzymes such as myeloperoxidase (MPO) and catalase, and triggers an oxidative response [2]. During this phase of post-STEMI inflammation, processes that worsen the detrimental effects of inflammation –particularly involving key cytokines (e.g., IL-1 and IL-6) and PMN-induced neutrophil extracellular trap (NET) formation– are especially active, further amplifying their harmful impact [3].

Local inflammation evolves into a systemic response characterized by elevated inflammatory biomarkers, making early pro-inflammatory markers valuable tools for risk stratification after STEMI. Among the biomarkers of inflammation in atherosclerotic cardiovascular disease, C-reactive protein (CRP) is the most extensively studied. CRP, an acute-phase protein, is predominantly produced by hepatocytes under the influence of cytokines such as IL-6 and tumor necrosis factor-α [4]. Elevated CRP levels are associated with an increased risk of atherosclerotic cardiovascular events. Some but not all studies have found that serum СRР predicts the risk of a recurrent in-hospital cardiac event [5] or 30-day or long-term mortality after STEMI [6,7].

Interleukin-1β amplifies inflammation by promoting its own expression in various cell types and stimulating the production of IL-6 [8]. Blocking IL-1β and IL-6 receptors with specific antibodies has been shown to reduce circulating CRP levels [9,10]. Elevated IL-6 levels are associated with an increased risk of major adverse cardiovascular events (MACE) following myocardial infarction [11].

On the other hand, emerging experimental and clinical evidence indicates that the formation of NETs in coronary thrombosis exerts harmful effects. NETs are modified chromatin structures released by neutrophils in response to specific stimuli, bound to cytoplasmic and granular proteins. Although NETs play a protective role against pathogens, dysregulated NETs exhibit strong proinflammatory and prothrombotic properties. In patients with STEMI, the presence of NETs in coronary thrombi is associated with larger infarcts and worse short-term outcomes [12–14]. The NET-specific circulating marker, citrullinated histone H3 DNA complex (H3Cit-DNA), plays a central role in NET formation. A standardized detection method is now available [15], although its clinical applicability in this context remains uncertain.

The primary objective of this study was to assess the utility of peripheral H3Cit-DNA levels and classical inflammatory markers in predicting adverse outcomes after STEMI.

## Patients and methods

We conducted a retrospective cohort study of patients with STEMI who underwent primary percutaneous coronary intervention (PPCI) between January 1, 2015 and January 5, 2019 in a tertiary academic hospital in Madrid. This project adhered to the Declaration of Helsinki and was approved by the Ethics Committee of our institution (Project ID 07.18). Data collection was based on a review of the participants’ medical records, and it was guaranteed that all persons involved in the study would respect the confidentiality of all patient information. Patients were identified by a numerical code to ensure the confidentiality of their personal data. The equivalence between the code and the patient was known only to the project coordinator. Data access date for research purposes was March 1, 2022.

### Patients

All patients with a final diagnosis of STEMI who presented to the hospital within 12 hours of symptom onset were consecutively included in the study. Patients who underwent more than one PPCI during the study period were included only once. All patients were of legal age and signed an informed consent form.

STEMI was defined as ST-segment elevation > 0.2 mV in 2 or more contiguous chest leads, > 0.1 mV in 2 or more limb leads, or new left bundle branch block on the electrocardiogram, combined with symptoms of myocardial ischemia and troponin I levels > 99th percentile. Diabetes mellitus was defined by previous diagnosis or random plasma glucose > 200 mg/dL on admission. Hypertension and dyslipidemia were defined by active drug treatment on admission or previous diagnosis. Previous ischemic heart disease was determined by history of myocardial infarction (MI), PCI, or coronary artery bypass grafting.

Epidemiologic, clinical, and angiographic data were collected from electronic medical records and coronary angiograms. Glycoprotein IIb/IIIa (GPIIbIIIa) inhibitors were administered at the discretion of the interventional cardiologist; their use was modified during the study period according to scientific evidence and guideline recommendations [16,17]. Patients were followed up routinely in the cardiology office or by a pre-consented telephone interview.

### Serum samples

Blood samples were collected immediately after PPCI and centrifuged at 3,500 rpm for 5 minutes. The separated serum was stored in aliquots at -80 °C in the biobank until analysis.

#### Analysis of H3Cit-DNA.

The H3Cit-DNA ELISA is described in detail here [15]. Briefly, calibration standards were prepared from H3R2,8,17 Cit dNucs (EpiCypher #16-1362) in a twofold dilution series at 1000, 500, 250, 125, 62.5, 31.3, 15.6 and 0 ng/mL in standard diluent (50 mmol/L Tris-HCl pH 7.5, 300 mmol/L NaCl, 0.01% [w/v] BSA, 0.01% [v/v] Tween-20). Uncoated high binding clear 96-well plates (Thermofisher #3855) were coated with an anti-H3R8Cit monoclonal capture antibody (Abcam ab232939). A horseradish peroxidase-conjugated anti-DNA antibody (Roche #11774425001, reconstituted according to the manufacturer’s instructions) was used as the detection antibody.

#### Detection of circulating double-stranded cell-free DNA (dsDNA).

The Quant-it PicoGreen dsDNA Kit (Invitrogen) and the Fluoroskan Ascent FL Kit with 485 nm (excitation) and 538 nm (emission) wavelength filters were used for dsDNA quantification.

#### Determination of serum levels of inflammatory markers.

The cytokines IL-6, TNFα, and IL-1β were determined by ELISA (Elabscience, China). The optical density (OD) was measured at 450 nm. The detection ranges were 15.63-1000 pg/mL for TNFα, 12.5-800 pg/mL for IL-6, and 31.25-2000 pg/mL for IL-1β. C-reactive protein was quantified by latex-enhanced immunoturbidimetric assay. The lower detection limit was 0.05 mg/dL. The main resources used in the analyses are listed online (S1 Table).

### Statistical analysis

Descriptive analysis was performed using mean and standard deviation or median and 25th and 75th percentiles, as appropriate. H3Cit-DNA was log2 transformed to obtain a log-normal distribution. Absolute and relative frequencies were used to describe categorical variables. Median follow-up was determined using the reverse Kaplan-Meier estimator. Survival functions were estimated by the Kaplan-Meier method.

Univariate analysis of the association between inflammatory markers and four clinical outcomes was performed using binary logistic regression (Killip classification 0/I vs II/III, 30-day MACE, and 30-day mortality after STEMI) or multinomial logistic regression (left ventricular ejection fraction [LVEF] < 35%; 35-50%; > 50%). In the logistic regression analysis, all inflammatory marker values were included, whether above or below the detection limit of the corresponding technique.

A prediction model for MACE at 30 days was developed using the following approach. First, all inflammatory markers were evaluated in a multivariable logistic regression model using an automated backward strategy based on p-values <0.05 to retain variables in the model. We refer to this as the ‘inflammatory marker model’. Second, we created another logistic model based on the classical risk factors: age, sex, smoking, hypertension, dyslipidemia, diabetes, previous ischemic heart disease, Killip class at admission, culprit artery of the infarction, affected arterial segment, number of coronary vessels with significant disease, TIMI classification after PPCI, and LVEF at discharge. Again, using an automated regression strategy based on p-values < 0.05, a “clinical model” was obtained. Finally, using a backward parsimonious strategy, a “clinical-inflammatory model” was developed by merging the two previous models. To assess whether the “clinical-inflammatory model” had a better discriminatory ability than the “clinical model”, the area under the receiver operating characteristic (AUC) was estimated for both models, as well as the reclassification ability by integrated discrimination improvement (IDI) and net reclassification improvement (NRI). We compared the models using a likelihood-ratio test.

A nomogram was developed for simplicity and applicability. Bootstrapping internal validation of the clinical-inflammatory model was performed with 500 resamples. Calibration was assessed using a calibration plot to provide calibration in the large (CITL) as well as slope. Discrimination was assessed using the c-statistic, which is equivalent to the area under the receiver operating characteristic (ROC) curve. The significance level was set at 0.05. Stata v. 18 was used for statistical analysis.

## Results

**Fig 1:**
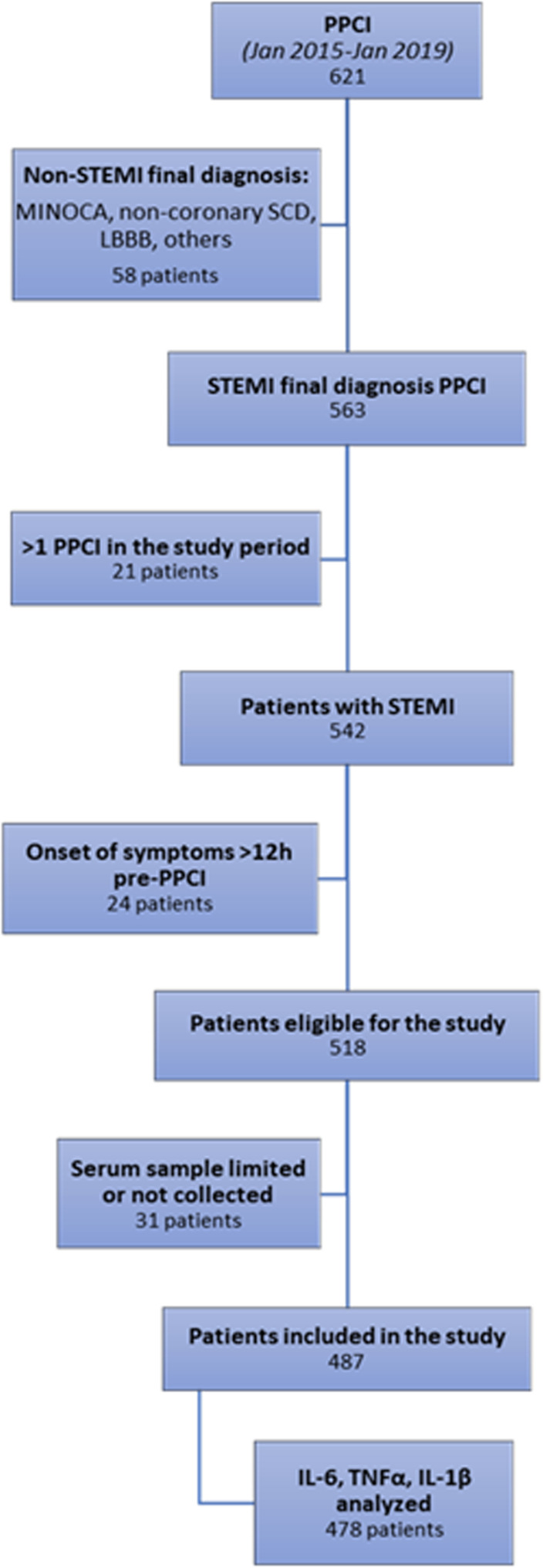
Flowchart of patients included in the study. IL-6, interleukin 6; IL-1β, interleukin 1 beta; LBBB, left bundle branch block, MINOCA, myocardial infarction and non‐obstructed coronary arteries; PPCI, primary percutaneous coronary intervention; SCD, sudden cardiac death; STEMI, ST-elevation myocardial infarction; TNFα, tumor necrosis factor alpha.

During the study period, 621 urgent coronary angiograms were performed, and the final diagnosis was STEMI in 563 cases. Patients who underwent more than one PPCI during the study period (n = 21) were included only once, and 24 patients admitted more than 12 hours after symptom onset were excluded. In 31 of the 518 eligible patients, the serum sample was insufficient, lost, or not collected at the time of PPCI. We performed a sensitivity analysis in this subgroup, and they are comparable to the remaining 487 patients in terms of baseline variables. Therefore, 487 patients were included in the study. A flow chart of the patients is shown in Fig 1.

The mean age of the patients was 63 (SD 13) years and 78% were male. A history of coronary artery disease was recorded in 14% of patients. Before admission, 13 patients (2.7%) were on oral anticoagulation, 98 (20%) were on single or dual antiplatelet therapy, and 160 (33%) were taking statins. Prior to PPCI, a loading dose of acetylsalicylic acid and a second antiplatelet agent were administered: clopidogrel (in 60.8% of patients), ticagrelor (23.0%), or prasugrel (14.5%). Fifty-three percent of patients received a gpIIb/IIIa inhibitor (eptifibatide) during and/or after the procedure. The majority (92%) were revascularized with at least one stent, which was a drug-eluting stent in 76% of cases. Table 1 summarizes baseline patient characteristics and serum levels of H3Cit-DNA and other inflammatory markers measured at the time of PPCI.

**Table 1:** Baseline characteristics of patients with STEMI.

		Total study sample (n = 487)
**Epidemiological and clinical features**		
Age, mean (SD), years		63 (13.1)
Male, no. (%)		380 (78.0)
BMI, mean (SD)		26.7 (24.6-29.6)
Hypertension, no. (%)		252 (51.8)
Smoking status^a^, no. (%)		
Current smoker		210 (43.1)
Non-smoker		277 (56.9)
Dyslipidemia, no. (%)		221 (45.4)
Diabetes, no. (%)		91 (18.7)
Ischemic cardiopathy, no. (%)		68 (14.0)
Chronic inflammatory/autoimmune disease, no. (%)		20 (4.1)
Immunological/anti-inflammatory treatment, no. (%)		29 (5.9)
Cancer history, no. (%)		46 (9.5)
Current cancer therapy, no. (%)		7 (1.4)
Killip status III or IV, no. (%)		49 (10.1)
Time symptom-balloon (min), median (IQR)		187.8 (139.1-271.8)
**Angiography, no. (%)**		
Culprit vessel		
RCA		199 (40.9)
LAD		208 (42.7)
Cx		70 (14.4)
Others		10 (2.1)
Arterial segment, proximal		195 (40.0)
Infarction due to stent thrombosis		33 (6.8)
Coronary vessels w. severe disease > 1		187 (38.4)
TIMI status after PCI II or III^b^		463 (95.1)
**Laboratory values on admission, median (IQR)**		
Leukocyte count, cells x10^3^/ µ L		12.1 (9.7-15.0)
Hemoglobin, g/dL		14.5 (13.2-15.7)
Platelet count, cells x10^3^/ µ L		236 (195-282)
Total cholesterol, mmol/L		173 (140-204)
LDL-cholesterol, mmol/L		105 (77-130)
Glucose, mmol/L		135 (116-168)
Peak troponin I, ng/L		66.4 (26.9-143.3)
GFR (CKD-EPI), ml/min/1.73 m2		83.4 (56.3-95.9)
**Inflammatory markers**	**Above detection limit** ^c^ **, no. (%)**	**Median (P25-75)** ^d^
H3CitDNA, ng/mL^e^	490 (100)	179.30 (105.30-281.47)
C-Reactive Protein, mg/L	391 (80.4)	2.96 (1.35-7.92)
Double-stranded DNA, ng/ml	490 (100)	503.26 (426.56-632.97)
Interleukin 6, pg/ml	198 (40.7)	9.16 (3.04-38.91)
TNF-α, pg/ml	59 (12.1)	3.98 (1.08-34.89)
Interleukin 1-β, pg/ml	39 (8.0)	7.81 (1.51-30.79)
**LVEF at hospital discharge, no. (%)**		
LVEF ≤ 35%		31 (6)
LVEF 35-50%		157 (32)

Abbreviations: BMI, body mass index; Cx, circumflex coronary artery; hs-cTnI, high-sensitive cardiac troponin I; DAPT, double antiplatelet therapy; GFR (CKD-EPI), glomerular filtration rate (Chronic Kidney Disease Epidemiology Collaboration); H3CitDNA, citrullinated histone-3 DNA; IQR, interquartile range; LAD, left anterior descending artery; LDL, low-density lipoprotein; LVEF, left ventricular ejection fraction; MI, myocardial infarction; PCI, percutaneous coronary intervention; RCA, right coronary artery; SD, standard deviation; ST, ST-segment on electrocardiogram; TIMI, Thrombolysis in Myocardial Infarction grade flow; TNF, tumor necrosis factor.

^a^Non-smoker refers to never smokers and ex-smokers for more than 1 month. Smokers refers to current smokers or former smokers for less than one month.

^b^TIMI grade flow: 0, no perfusion; I, penetration without perfusion; II, partial perfusion; and III, complete perfusion.

^c^Refers to the number (%) of test results with detectable levels by the corresponding laboratory method.

^d^Refers to the median and the 25th and 75th percentiles of the values detectable with the corresponding method of analysis.

^e^Log2 transformation of H3Cit-DNA to obtain log normal distribution, median (P25-75): 7.49 (6.72-8.14)

Median (95% CI) follow-up was 5.4 (5.2-5.5) years. The proportion of patients with MACE after STEMI was 6.4% (31 patients/n = 485) at 1 month, 14.4% (70/485) at 1 year and 17.9% (87/485) at 2 years. Mortality was 4.1% (20/487) at 1 month, 6.6% (32/487) at 1 year, and 7.8% (38/487) at 2 years. The adverse events recorded during the first month after STEMI included 20 deaths, 5 cases of re-STEMI, and 6 episodes of heart failure requiring hospitalization. During this period, all deaths were cardiac-related, and no patients were lost to follow-up.

Table 2 shows the univariable analysis of the association between inflammatory markers and the four clinical endpoints (Killip classification, LVEF and MACE, and 30-day mortality after STEMI). Serum H3Cit-DNA levels (median [IQR] 179.30 [105.30-281.47] ng/mL) were not associated with any outcomes, except for a mild to moderate reduction in LVEF (35–50%) compared to normal LVEF (reference category: > 50%). The relative risk ratios (RRR) were as follows: < 35%, 1.01 (95% CI, 0.74–1.38; p = 0.952); 35–50%, 1.26 (95% CI, 1.07–1.48; p = 0.005). CRP, IL-6, TNFα, and IL-1β were detectable in 80%, 41%, 12%, and 8% of patients, respectively (Table 1). C-reactive protein and IL-6 levels were significantly associated with all four clinical endpoints in univariable analysis. Both dsDNA and TNFα were significantly associated with 30-day mortality, Killip class, and 30-day MACE.

**Table 2:** Univariable analysis of inflammation markers and Killip classification, LVEF, 30-day MACE and mortality post-STEMI.

	Killip status on admission		LVEF at discharge (>50% as reference)				
	OR (95% CI)	*p*-value	RRR (95% CI) < 35%	*p*-value	RRR (95% CI) 35-50%		*p*-value
CRP^a^, per 10 mg/L increase	1.11 (1.02-1.21)	0.017	1.04 (0.85-1.27)	0.711	1.14 (1.04-1.25)		0.004
IL 6^b^, per 10 pg/mL increase (^*^)	1.43 (1.30-1.57)	<0.001	1.26 (1.12-1.42)	<0.001	1.10 (1.01-1.20)		0.035
Leukocyte count, cells x10^3^/ µ L	1.14 (1.07-1.21)	0.001	1.14 (1.05-1.23)	0.001	1.05 (1.00-1.10)		0.048
dsDNA, per 50 ng/mL increase	1.03 (1.01-1.05)	<0.001	0.98 (0.92-1.04)	0.461	1.01 (1.00-1.02)		0.187
TNFα, per 50 pg/mL increase	1.10 (0.82-1.46)	0.528	0.77 (0.29-2.02)	0.593	0.82 (0.56-1.20)		0.310
IL-1β, per 10 pg/mL increase	0.89 (0.57-1.39)	0.612	0.97 (0.62-1.51)	0.886	1.01 (0.84-1.23)		0.897
H3Cit-DNA Log2, ng/mL	0.95 (0.74-1.21)	0.664	1.01 (0.74-1.38)	0.952	1.26 (1.07-1.48)		0.005
	**30-day MACE**			**30-day mortality**			
	**OR (95% CI)**		*p* **-value**	**OR (95% CI)**		*p* **- value**	
CRP^a^, per 10 mg/L increase	1.26 (1.15-1.37)		<0.001	1.24 (1.13-1.36)		<0.001	
IL-6^b^, per 10 pg/mL increase	1.39 (1.26-1.54)		<0.001	1.43 (1.26-1.61)		<0.001	
Leukocyte count, cells x10^3^/ µ L	1.08 (1.00-1.17)		0.041	1.19 (1.09-1.31)		<0.001	
dsDNA, per 50 ng/mL increase	1.03 (1.01-1.05)		0.001	1.03 (1.01-1.05)		<0.001	
TNFα, per 50 pg/mL increase	1.36 (1.07-1.73)		0.012	1.46 (1.14-1.86)		0.003	
IL-1β, per 10 pg/mL increase	1.14 (0.92-1.42)		0.236	1.16 (0.91-1.49)		0.225	
H3Cit-DNA Log2, ng/mL	0.98 (0.72-1.32)		0.879	1.03 (0.71-1.50)		0.861	

Abbreviations: CRP, C-reactive protein; dsDNA, double-stranded DNA; H3CitDNA, citrullinated histone-3 DNA; IL, interleukin; LVEF, left ventricular ejection fraction; OR, odds ratio; RRR, relative risk reduction; TNF, tumor necrosis factor

^a^^,^^b^ Selected variables for the inflammatory markers model

The “inflammatory marker model” incorporated CRP and IL-6 to predict 30-day MACE. The initial variables included in the clinical model were age, sex, smoking status, hypertension, dyslipidemia, diabetes, previous ischemic heart disease (IHD), Killip class on admission, TIMI flow after PCI, number of diseased vessels, the culprit coronary artery and segment affected, and LVEF at discharge. After applying a backward selection strategy, the final clinical model for 30-day MACE included previous IHD, Killip class, final TIMI flow, and hypertension. The “clinical-inflammatory model” was developed by combining the final clinical and inflammatory marker models. Results of the 30-day analysis are presented in Table 3.

**Table 3:** Multivariable clinical-inflammatory model for the appearance of major cardiovascular events 30 days after STEMI.

	Clinical-inflammatory markers model	
	OR (95% CI)	*p*-value
Age (years)	1.05 (1.01-1.09)	0.007
Killip status, III or IV	5.02 (1.68-15.05)	0.004
TIMI after PCI, II or III	0.24 (0.65-0.88)	0.032
C-reactive protein (mg/L)	1.02 (1.01-1.03)	0.001
IL-6, per 10 pg/mL increase	1.02 (1.01-1.03)	0.007

Abbreviations: CI, confidence interval; IL, interleukin; OR, odds ratio; STEMI, ST-elevation myocardial infarction; TIMI, Thrombolysis in Myocardial Infarction grade flow.

**Fig 2:**
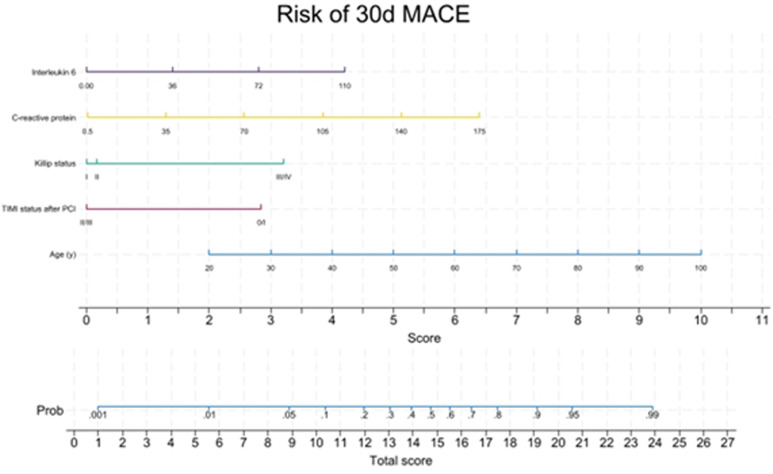
Nomogram of the risk of major cardiovascular events 30 days post-STEMI.

At 30 days, the addition of CRP and IL-6 significantly enhanced the clinical model’s performance (likelihood-ratio test p < 0.0001; AUC 0.866 vs. 0.884; NRI 31.7% and IDI 13%). The nomogram for the 30-day MACE prediction model is shown in Fig 2.

In terms of internal validation, the CITL and slopes after bootstrapping were 0.005 (95%CI -0.471; 0.546) and 0.906 (95%CI 0.601; 1.186) for the 30-day MACE model. The optimism-adjusted c-statistic was 0.873 (95%CI 0.798; 0.943). Calibration plot of 30-day MACE prediction model shown as supplemental data (S1 Fig).

## Discussion

This study evaluated the short-term prognostic value of classical inflammatory markers and the NET-specific marker H3Cit-DNA within 30 days after STEMI. To our surprise, we found no association between H3Cit-DNA and adverse outcomes after STEMI. Additionally, H3Cit-DNA levels were not associated with intermediate variables, such as admission Killip class or discharge LVEF. Instead, C-reactive protein and interleukin 6 significantly enhanced the discriminative ability of a 30-day STEMI clinical risk prediction model.

NETs in coronary thrombi from patients with STEMI [12,18–21] have been directly related to infarct size and inversely related to ST-segment resolution [12]. More recently, NET formation in coronary thrombi has also been associated with patient outcome after STEMI [13]. Moreover, Hofbauer et al [14] found a remarkable local increase of monocyte chemoattractant protein (MCP)-1 and NET markers [dsDNA and H3Cit] in the culprit artery of the infarct and described a mutual induction of NETs and MCP-1, which in turn was associated with recurrent cardiovascular events [22] and all-cause mortality after myocardial infarction[23].

Our group demonstrated a high NET burden in coronary thrombi of a small series of patients with COVID-19 [24], suggesting that NETs play a pivotal role in coronary thrombosis in severe SARS-CoV-2 infection, similar to what has been demonstrated in other organs. Thus, this condition may be paradigmatic for an inflammatory state predisposing to STEMI.

Therefore, finding a reliable circulating NET marker in patients with STEMI seems to be a noteworthy goal and has been pursued by several investigators [25–27]. There is evidence of an association between post-infarction dsDNA levels and the occurrence of adverse events after STEMI, but dsDNA is a non-specific marker of NETosis, which is also associated with cell destruction. In contrast, this association is not clear for NET-specific markers. In the largest series (259 patients), Langseth et al [25] found a direct association between dsDNA levels and the occurrence of adverse clinical events one year after STEMI; such an association was not observed for two NET-specific markers, MPO–DNA complex and H3Cit. In this study, serum samples were collected at a median of 18 hours following PCI.

In a smaller series [26] involving 83 patients with similar characteristics, dsDNA, MPO-DNA complex, and NETs-related tissue factor were analyzed in patients undergoing PCI within the first 12 hours after STEMI. Plasma samples were collected from the infarct-related artery (IRA) and the radial artery during PCI. All three markers were significantly higher in samples obtained from the IRA compared to peripheral arteries. Conversely, no significant difference was observed in TNFα levels between coronary and peripheral blood. Only coronary dsDNA was independently associated with the development of in-hospital MACE.

Until recently, determination of H3Cit in peripheral blood has been unreliable. Thålin et al [15] have shown that the detection of enzymatically citrullinated H3 proteins is hampered by large enzyme-dependent batch variability as well as their instability in plasma, and that most commercially available antibodies against intrapeptidyl citrulline have poor specificity for the described target. These investigators have recently developed a new assay using highly specific monoclonal antibodies and semisynthetic nucleosomes containing citrulline instead of arginine at histone H3, arginine residues 2, 8 and 17 (H3R2,8,17Cit) as calibration standards. Validation of the assay demonstrated its ability to accurately and reliably quantify H3Cit-DNA levels in human plasma.

The lack of association between circulating H3Cit levels and outcomes after STEMI in previous studies may be due to the aforementioned technical issues. Similarly, a recent study [28] raised concerns about the detection of circulating MPO-DNA complexes, noting that the specificity of commercial ELISA kits for NET detection is “highly questionable.”

Recently, Benkhoff et al. [29] studied 361 patients with STEMI and reported an association between H3Cit-DNA levels in plasma samples collected 24 hours after presentation and MACE at 12 months. H3Cit-DNA levels were also linked to early mortality (within 30 days). This study employed the standardized method of Thålin et al. [15]. A key distinction compared to our study, which may explain these findings, is the cohort’s very high cardiovascular risk profile prior to STEMI –64% of patients had hypertension and 40% had diabetes– and a remarkably high early mortality rate of approximately 20%. Regarding H3Cit-DNA kinetics post-STEMI, Benkhoff [29] observed that levels remained relatively stable during the first few days, suggesting a persistent thromboinflammatory state. Another study [30] reported that NET markers increased significantly 30 minutes after PCI, decreased at 24 hours, but remained elevated compared to the control group.

The present study assessed inflammatory markers for their ability to predict MACE in the short term following myocardial infarction. In addition to H3Cit-DNA, the analyzed markers included dsDNA, CRP, IL-6, TNFα, and IL-1β. There is clinical evidence supporting the prognostic value of CRP and IL-6 in acute coronary syndrome; however, the relevance of IL-1β and TNF-α as prognostic markers in this context remains uncertain [3]. Our findings confirm the prognostic value of CPR and IL-6 after STEMI. In our series, CRP and IL-6 levels were above the detection threshold in 80% and 41% of patients, respectively; whereas TNFα and IL-1β were detectable in only 12% and 8% of patients. The prognostic capacity of these markers likely reflects their expression in peripheral blood post-STEMI. The low detectability of TNFα and IL-1β highlights their limited utility as prognostic indicators in this setting.

At 30 days, a model combining three clinical parameters –age, Killip class, and TIMI flow after PPCI– with CRP and IL-6 levels demonstrated excellent prognostic discriminative ability. The inclusion of CRP and IL-6 significantly enhanced the predictive performance of the clinical model by the likelihood-ratio test. Additionally, the net reclassification improvement (NRI) of 31.7% indicates that a significant proportion of individuals had their risk categories more accurately classified. The integrated discrimination improvement (IDI) of 13% further highlights the model’s enhanced ability to distinguish between individuals with and without the outcome. These findings underscore the clinical utility of incorporating CRP and IL-6 into the model, emphasizing their potential to improve risk stratification and guide decision-making.

C-reactive protein is a widely used marker of inflammation. The widespread use of IL-6 during the SARS-CoV-2 pandemic may facilitate its incorporation into clinical practice to select, together with CRP, patients with STEMI who may benefit from inflammation-targeted therapies in the future.

This study has several limitations. Firstly, H3Cit is part of the PAD4-dependent NET formation pathway [31] and we would thus not quantify PAD4-independent NET formation. However, it should be noted that H3Cit is a central player in neutrophil nuclear chromatin release and its circulating levels are useful for predicting mortality in cancer patients [32]. Second, the H3Cit-DNA ELISA was developed and optimized for quantification in citrated plasma and, although it has previously been applied on serum samples [33], we cannot exclude suboptimal performance in this medium. Lastly, this is a single-center retrospective cohort study with a limited sample size and a low rate of MACE, and external testing on larger series would be necessary to confirm our results.

## Conclusions

Circulating levels of the NET marker H3Cit-DNA were not associated with cardiovascular events after STEMI. Instead, C-reactive protein and interleukin-6 were associated with MACE and significantly improved the predictive ability of a clinical 30-day risk prediction model after STEMI.

## Supporting information

S1 Fig Calibration plots of the prediction model at 1 month.
(TIF)

S1 Table Key resources.
(DOCX)
