# Supplementary figures and images for: Inflammatory and neutrophil extracellular trap markers to predict cardiac events after ST-segment elevation myocardial infarction
Source: PLoS One. 2025 Apr 1;20(4):e0319759. doi: 10.1371/journal.pone.0319759 (PMC11960995; doi:10.1371/journal.pone.0319759)

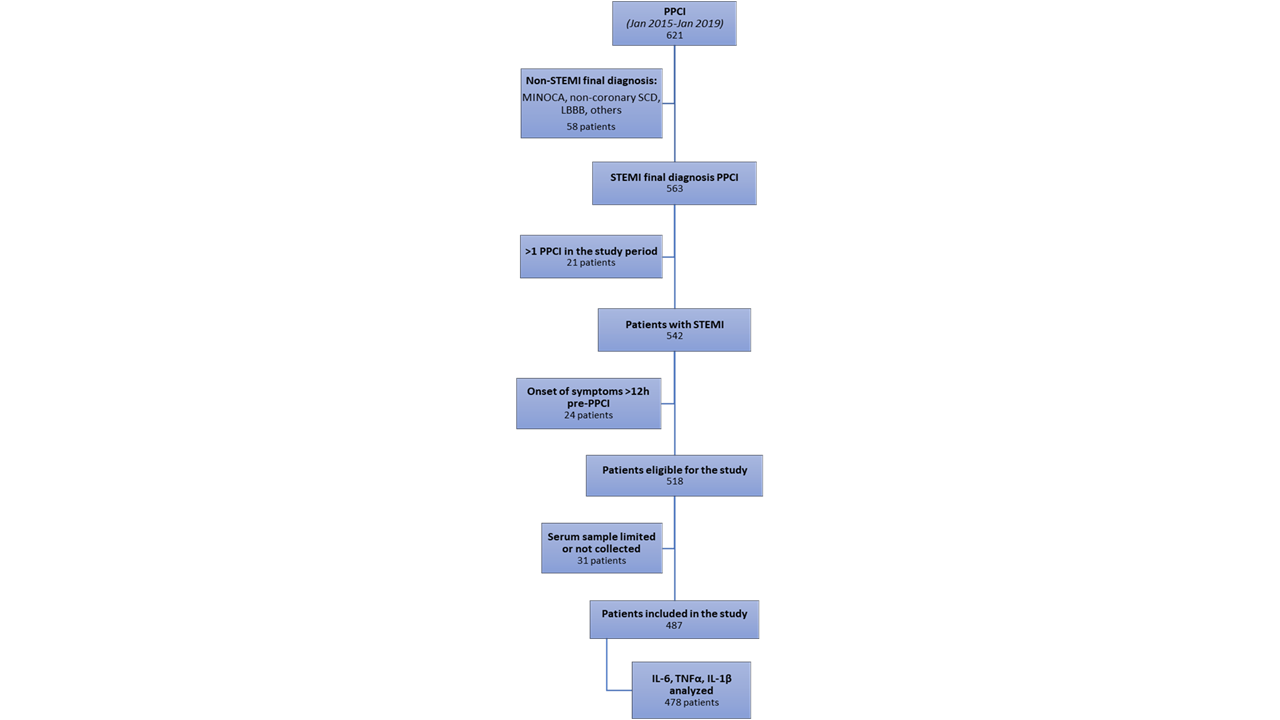

Supplement: S1 Fig — Calibration plots of the prediction model at 1 month. (TIF) [file pone.0319759.s001.tif]
