# Supplementary material for: Inflammatory and neutrophil extracellular trap markers to predict cardiac events after ST-segment elevation myocardial infarction
Source: PLoS One. 2025 Apr 1;20(4):e0319759. doi: 10.1371/journal.pone.0319759 (PMC11960995; doi:10.1371/journal.pone.0319759)
Supplement: S1 Table — (DOCX) [file pone.0319759.s002.docx]

**Supplementary Table. Key resources**

| **Reagent type** | **Source** | **Identifier** |
| --- | --- | --- |
| High Bind Clear 96-well microplates | Thermo Fisher Scientific | 3855 |
| H3Cit ELISA PLUS | Roche | 11 774425001 |
| H3R2,8,17 Cit dNucs (EpiCypher #16-1362) | EpiCypher | 16-1362 |
| Horseradish peroxidase (HRP) substrate (TFS #34028) | Thermo Fisher Scientific | 34028 |
| IL6, TNF, IL1 ELISA | Elabscience, China | [D6050B](https://www.rndsystems.com/products/human-il-6-quantikine-elisa-kit_d6050b)  [201-LB](https://www.rndsystems.com/products/recombinant-human-il-1-beta-il-1f2-protein_201-lb)  [DTA00D](https://www.rndsystems.com/products/human-tnf-alpha-quantikine-elisa-kit_dta00d) |
| Quant-it PicoGreen dsDNA | Thermo Fisher Scientific | P11496 |
| TRIS-HCL | Sigma(no se cual han usado) | 1185-53-1 |
| 96 well plates for fluorescence-1 box | Thermo Fisher Scientific | M33089 |
